# Ocular Manifestations of Patients with Coronavirus Disease 2019: A Comprehensive Review

**DOI:** 10.18502/jovr.v16i2.9087

**Published:** 2021-04-29

**Authors:** Amirhossein Roshanshad, Mohammad Ali Ashraf, Romina Roshanshad, Ali Kharmandar, Seyed Alireza Zomorodian, Hossein Ashraf

**Affiliations:** ^1^Student Research Committee, Shiraz University of Medical Sciences, Shiraz, Iran; ^2^Poostchi Ophthalmology Research Center, Shiraz University of Medical Sciences, Shiraz, Iran; ^3^Non-communicable Disease Research Center, Fasa University of Medical Sciences, Fasa, Iran; ^4^Health Policy Research Center, Institute of Health, Shiraz University of Medical Sciences, Shiraz, Iran

**Keywords:** Coronavirus, COVID-19, Manifestations, Ocular, Ophthalmologic

## Abstract

Apart from conjunctival involvement which is the most well-known ocular manifestation of coronavirus infectious disease 2019 (COVID-19), there are multiple reports of the involvement of other ocular structures by severe acute respiratory syndrome coronavirus 2 (SARS-CoV-2). We comprehensively reviewed PubMed, Scopus, Embase, and Google Scholar for available evidence regarding COVID-19 various ocular manifestations, with special focus on less known and unusual ocular findings. We then categorized the findings based on the parts of the eye which was involved. In anterior sections of the eye, the involvement of the eyelid (tarsadenitis), conjunctiva and cornea (follicular conjunctivitis, pseudomembranous conjunctivitis, and keratoconjunctivitis), episclera (nodular episcleritis), uvea (anterior uveitis) were reported. Also, third, fourth, and sixth nerve palsy, retinal vasculitis, retinal optical coherence tomography (OCT) changes (hyper-reflective lesions and increased retinal nerve fiber layer thickness [RNFLT]), optic neuritis, papillophlebitis, Miller Fisher syndrome, posterior reversible leukoencephalopathy (PRES), ophthalmic artery and central retinal artery occlusion, and polyneuritis cranialis were reported in different studies. Postmortem evaluation of COVID-19 patients detected no viral RNA in different anterior and posterior segments of the eyes. However, another study revealed a 21.4% positivity of the retinal biopsies of dead patients. The results of this study can help ophthalmologists to be vigilant when they see these findings in a suspected case of COVID-19. In addition, wearing face masks and protective goggles or eye shields are recommended, especially in high risk contacts.

##  INTRODUCTION

Since December 2019, coronavirus has caused more than 990,000 deaths and contamination of more than 32.7 million people globally till September 27, 2020.^[1]^ Coronavirus disease 2019 (COVID-19) can be transmitted directly through person to person contacts via droplets released during sneezing or coughing. Indirect transmission of severe acute respiratory syndrome coronavirus 2 (SARS-CoV-2) through surface contamination can be regarded as the second way of getting infected.^[2,3,4]^ In addition, SARS-CoV-2 has been detected in tears and conjunctival secretions of infected people.^[5]^ Therefore, adhering to personal hygiene principles is a fundamental part of COVID-19 prevention, especially for healthcare workers.

Ophthalmologists are at high risk of contracting COVID-19 due to their close contact with the patients during routine ophthalmologic exams such as slit lamp examinations and direct ophthalmoscopy.^[6]^ Also, the patients' prolonged stay at ophthalmology clinics for multiple ophthalmology examinations imposes ophthalmologists and other patients at increased risk of COVID-19.^[7]^ A recent study revealed a high probability of contamination of environmental surfaces of ophthalmology clinics.^[8]^ Ocular presentations of COVID-19 usually occur about two weeks after the first symptoms. However, it can be the presenting finding of newly diagnosed COVID-19 patients, especially when the virus enters from the eye mucosa.^[9]^ Consequently, knowing the latest presentations of COVID-19 is crucial for every ophthalmologist. It helps ophthalmologists to consider COVID-19 as a possible causative agent when they see these findings.

Like SARS-CoV, angiotensin-converting enzyme 2 (ACE 2) receptor is the mediator of SARS-CoV-2 for entering the host cells.^[10,11,12]^ ACE 2 receptor is expressed in different human tissue, including the pulmonary system, proximal tubule of kidney and bladder urothelium, myocardial cells, esophagus, and ileum.^[13]^ In the eye, ACE 2 receptor expression is reported in the conjunctiva, limbus, cornea, retina, and aqueous humor.^[14,15,16,17]^ It was thought earlier that COVID-19 only involves conjunctiva, cornea, and tear. However, recent studies have revealed the involvement of the other eye structures, such as eyelid, episcleral, and retina. Therefore, we gathered available data about ocular manifestations of COVID-19, focusing on recently reported manifestations.

##  METHODS

We systematically searched four databases, including Medline (Pubmed), Scopus, Embase, and Google Scholar for articles about ocular manifestations of COVID-19. We used the following keywords for searching the databases: (“coronavirus” OR “covid-19” OR “2019-ncov” OR “sars-cov-2”) AND (“ocular” OR “eye” OR “cornea” OR “conjunctivitis” OR “conjunctiva” OR “conjunctival” OR “congestion” OR “uvea” OR “uveitis” OR “retina” OR “retinal” OR “retinitis” OR “optic” OR “lens” OR “chemosis” OR “blepharitis” OR “ophthalmic” OR “ophthalmologic” OR “ophthalmoplegia” OR “ophthalmoparesis” OR “nerve palsy”). We also searched the references of the included studies for more relevant articles. The primary search was done on June 19, 2020 and was updated on August 27, 2020. The inclusion criteria were original articles about the ocular manifestations of COVID-19 patients. Case reports and case series, letters, and editorials were also included. Studies without any information about ocular manifestations of COVID-19 were excluded. Also, some of the studies reporting duplicate and repeated well-known ocular manifestations of COVID-19 were excluded, and the main focus was put on the less reported ophthalmologic presentations. Included studies were categorized in this study and divided based on the involved part of the eye.

We used Joanna Briggs Institute (JBI)'s critical appraisal tool^[18]^ to assess the risk of bias of the included studies. The scores were calculated as percentages and studies with scores of >60% and <30% of total scores were regarded as low- and high risk of bias, respectively. Title and abstract screening, data extraction and risk of bias assessment were done by two authors (AR and MA) independently and disagreements were checked by another author (RR).

**Figure 1:**
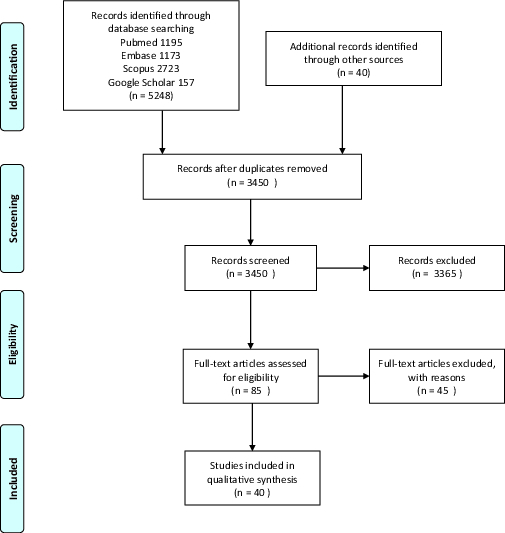
PRISMA flow chart of the screened and assessed articles.

As shown in Figure 1, a total of 5,288 studies were initially found after searching the databases according to the aforementioned search strategy. After title and abstract screening, 85 studies were chosen for full-text evaluation. Finally, 40 studies were matched with our inclusion criteria to enter the study. Conjunctival involvement and conjunctival and tear COVID-19 PCR positivity have been reported before. Furthermore, there are several reports of some rare ocular manifestations of COVID-19, which are unfamiliar to many ophthalmologists and physicians. Therefore, we summarized the conjunctival findings part in our study and focused more on the less reported and less known ocular manifestations. The findings of each part of the eye are discussed separately.

### Ocular Manifestations

#### Eyelid

A woman from Wuhan, China, who presented with lower eyelid swelling, pain, and tenderness in the lateral canthus of the right eye was diagnosed as acute tarsadenitis. The patient had visited her parents who had tested positive for COVID-19 one day before the onset of these manifestations. Along with the resolution of tarsadenitis, the patient developed subconjunctival hemorrhage. Radiologic and PCR test results were in favor of COVID-19 infection.^[19]^Another study reported multiple cases of chalazion in three ICU nurses and ten additional cases in a hospital in Paris.^[20]^ PCR results of the ICU nurses were negative. Because of the role of bacterial infection in eyelid disorders such as blepharitis and meibomian gland diseases,^[21,22]^ it is far from the mind that SARS-CoV-2 itself could be responsible for the observed tarsadenitis and chalazion. However, it is thought that the cluster of chalazion was mainly due to the occupational conditions while managing COVID-19 patients. Firstly, prolonged use of eye goggles and the following reduced eye blinking can facilitate the evaporation of the tear film and hardening of the meibomian gland secretions. Secondly, the use of some disinfectants for cleaning eye shields can be irritating for the eyes.^[20]^ Thirdly, due to the role of blood supply on fat synthesis, we hypothesize that impaired blood flow due to the hypercoagulable state in COVID-19 patients^[23]^ can change the composition of meibomian gland secretions and predispose the eyes to bacterial infection and following meibomian gland infection and tarsadenitis.^[24]^ Summary of the findings of eyelid, ocular surface, uvea, episclera, and ocular motor nerves are presented in Table 1.

#### Ocular surface: conjunctiva and cornea

Hyperemia, epiphora, increased secretion, chemosis, and follicular conjunctivitis are the main symptoms of conjunctival involvement with SARS-CoV-2.^[25]^ Based on the “Report of the WHO-China Joint Mission on Coronavirus Disease 2019 (COVID-19),” conjunctival congestion occurs in about 0.8% of COVID-19 patients.^[26]^ However, other studies showed higher rates. In Chen et al study, about 5% of COVID-19 patients had conjunctival congestions, 15% of whom as the initial presentations. Another study showed that 3.1% of COVID-19 patients presented with conjunctivitis, 0.7% of whom as the first symptom of the disease.^[27]^ A high prevalence of conjunctivitis was reported to be 32% in Wu et al's study.^[9]^ A meta-analysis stated that the overall prevalence of conjunctivitis among COVID-19 patients is about 1.1%. It also showed that this prevalence was 0.7% in non-severe cases and 3% in severe ones.^[28]^ Common ocular symptoms of conjunctival congestion are increased conjunctival secretion, ocular pain, photophobia, dry eye, and tearing. It was also shown that conjunctival congestion is higher in patients with more hand–eye contact.^[29]^

In another study, a patient presented with conjunctival hyperemia, secretion, follicles, petechiae, tarsal hemorrhages, and chemosis with easily removed yellowish membrane on the tarsal conjunctiva of the lower lids. He was diagnosed as pseudomembranous conjunctivitis and punctate keratitis.^[30]^

Positive SARS-CoV-2 detection in conjunctival samples or ocular secretions has been reported in several studies.^[31,32]^ A systematic review showed that conjunctival/tear PCR samples were positive in about 2% of COVID-19 patients.^[27]^ Different studies revealed variation in positive PCR rate from 3 to 16%, with an average of 5.8%.^[33]^

These variations may be due to the technical problems during collecting, keeping, and handling of the specimen, low viral load in the conjunctival secretion of some participants, and also the difference between the severity of the disease in different studies.^[34,35]^

Reminding the experience with SARS-CoV-1 increased transmission during unprotected eye contacts,^[36]^ and possible transmission of SARS-CoV-2 through the conjunctiva, wearing protective goggles should become routine among healthcare workers with high risk contacts.^[37]^ It should be kept in mind that keratoconjunctivitis can be the initial manifestation of patients with COVID-19 infection.^[38]^ Therefore, first-line healthcare providers should ask questions regarding other manifestations of COVID-19 like fever and cough and a history of suspicious contact in patients presenting with a red eye. It is also revealed that conjunctival/tear PCR samples may be positive in some patients despite not having any ocular manifestations.^[27]^ It prompts the need for adherence to protective measures and wearing personal protective equipment even if no clinical symptoms are observed.

**Table 1:** Characteristics and main findings of the included studies (eyelid, ocular surface, episcleral, uveal, and ocular motor nerve findings)


First author${}^{*}$	**Date of publication**	**Number of cases**	**Risk of Bias**	**Ocular manifestations or findings**	**Additional findings/Remarks**	**Diagnosis**
**Eyelid**						
Xu^[19]^	April 2020	1	Medium	Pain in the lateral canthus and lower eyelid swelling	The same as symptoms	Tarsadenitis
Megarbane${}^{†}$ ^[20]^	May 2020	3	High	Red eye, painless eyelid swelling, and tearing in three cases	Single non-tender inflammatory nodules in the middle of the lower eyelid, conjunctival redness without altered visual acuity or corneal abrasion	Chalazion
**Conjunctiva and cornea**						
Bostanci Ceran^[25]^	June 2020	93	Low	21% of the patients had ocular problems. Photophobia (16.1%), itchiness (15.7%), burning sensation (8.4%), gritty feeling (6%), blurred vision (4.8%)	Hyperemia (21.5%), epiphora (9.7%), increased secretion (6.5%)	Chemosis (3.2%), follicular conjunctivitis (8.6)
WHO^[26]^	February 2020	55,924	N/A	0.8% of the patients had conjunctival congestion	
Loffredo^[28]^	April 2020	1,167	Medium	Overall rate of conjunctivitis was 1.1%; 0.7% in non-severe cases and 3% in severe cases	
Chen^[29]^	May 2020	535	High	Increased conjunctival secretion (9.7%), ocular pain (4.3%), photophobia (3%), dry eye (21%) and tearing (10.3%)	5% had conjunctival congestion, 15% of whom as the initial finding. Other findings were: conjunctivitis (6.2%), xerophthalmia (4.5%), and keratitis (2.6%)	
Navel^[30]^	May 2020	1	Low	Follicles, petechias, tarsal hemorrhages, and chemosis	Mucous filaments and tarsal pseudomembranous	Pseudomembranous conjunctivitis
Cheema^[38]^	April 2020	1	Low	Red eye, watery discharge, photophobia	Conjunctival injection, follicles, pseudodendrite in the cornea, and subepithelial infiltrates with overlying epithelial defects at the limbus	Keratoconjunctivitis
Wu^[9]^	38	High	31.6% had ocular manifestations consistent with conjunctivitis including hyperemia (7.9%), chemosis (21%), epiphora (18.4%), or increased secretions (18.4%)	
Sarma^[27]^	April 2020	854	Low	3.2% had conjunctivitis/red eye, 0.7% reported conjunctivitis as the first symptom of the disease. 2% positive conjunctival/tear PCR samples	
Chen^[31]^	April 2020	1	Low	Positivity of conjunctival swab specimens till 19 days after the disease onset	Conjunctivitis
Colavita^[32]^	August 2020	1	Medium	Higher viral load in late ocular samples than nasal swabs	Positivity of ocular samples till 21 days after the disease onset	Conjunctivitis
Aiello^[33]^	May 2020	252	Medium	12/204 (5.8%) had positive PCR of conjunctival swab	
**Episclera**						
Mendez Mangana^[39]^	June 2020	1	Low	Red eye, foreign-body sensation, epiphora, and photophobia	Elevated epibulbar area with hyperemia at the inferotemporal sector without fluorescein defect	Nodular episcleritis
Bostanci Ceran^[25]^	June 2020	93	Low	2.2% of the patients had episcleritis	Episcleritis
**Uvea and Glaucoma**						
Bettach^[40]^	June 2020	1	Low	Bilateral blurry vision	Conjunctival hyperemia, central corneal edema with Descemet's membrane folds, multiple keratic precipitates, and +1 anterior chamber cells and flare	Anterior uveitis
**Ocular motor nerve**						
Dinkin^[49]^	May 2020	2	High	Case 1: ptosis, diplopia Case 2: diplopia, abduction deficit	Case 1: mydriasis, ptosis and limited depression, adduction, and abduction Case 2: enhancement of the optic nerve sheaths and posterior Tenon capsules	Case 1: third and sixth nerve palsy Case 2: optic nerve involvement
Falcone^[52]^	June 2020	1	Medium	Binocular diplopia	Esotropia and limitation of abduction of the left eye. Atrophic left lateral rectus muscle in MRI	Sixth nerve palsy
de Oliveira^[53]^	June 2020	1	Low	Binocular diplopia and occipital headache	MRI showed vertebrobasilar system vasculitis, inflammation in the periaqueductal region and trochlear nuclei topography	Fourth nerve palsy
*The studies are categorized based on the section of the eye involved in a study ${}^{†}$In this study, participants were three nurses working in a COVID-19 ICU but had negative PCR test results N/A, Not available						

**Table 2:** Characteristics and main findings of the included studies (retina, neuro–ophthalmology, thromboembolic, and postmortem findings)


First author^*^	**Date of publication**	**Number of cases**	**Risk of Bias**	**Ocular manifestations or findings**	**Additional findings/Remarks**	**Diagnosis**
**Retina**						
Casagrande^[55]^	May 2020	14	Medium	Positivity of retinal biopsy samples in 3 of 14 (21.4%) COVID-19 patients	
Marinho^[56]^	May 2020	12	High	Hyper-reflective lesions in ganglion cell and inner plexiform layers more obvious at the papillomacular bundle, normal OCT-angiography and ganglion cells complex analysis in all patients, cotton wool spots and microhemorrhages in four (33.3%) patients	
Quintana-Castanedo^[59]^	July 2020	1	High	Visually asymptomatic	Retinal vasculitis on the equator of the left eye, one perivascular infiltrate, and extended retinal exudates	Retinal vasculitis
Burgos-Blasco^[60]^	July 2020	5	Medium	Visually asymptomatic	Seven out of eight (87.5%) eyes showed an increase in RNFLT	
Insausti-García^[61]^	July 2020	1	Low	Reduced sensitivity of the visual field in left eye	Tortuous and dilated retinal vessels, retinal hemorrhages, and disc edema	Papillo-phlebitis
Raony^[62]^	June 2020	0	N/A	Cytokine storm can aggravate retinal lesions in infected patients with DM. CD-147 may also facilitate retinal invasion of SARS-CoV-2	
**Neuro-ophthalmology**						
Gutiérrez-Ortiz^[70]^	April 2020	2	Medium	Case 1: right INO and fascicular oculomotor palsy Case 2: bilateral abducens palsy	Case 1: albuminocytologic dissociation, positive GD1b-IgG antibodies Case 2: albuminocytologic dissociation	Case 1:MFS Case 2: polyneuritis cranialis
Lantos^[71]^	May 2020	1	Low	Left eye drooping, blurry vision, reduced sensation in both legs, ataxia	Third cranial nerve enhancement and enlargement in MRI consistent with third nerve palsy, bilateral sixth nerve palsy decreased sensation below the knees to all modalities	MFS
Kaya^[73]^	April 2020	1	Medium	Acute confusional state, vision loss	MRI showed vasogenic edema similar to posterior reversible leukoencephalopathy (PRES)	PRES
Doo^†^^[74]^	July 2020	2	High	Visually asymptomatic	Bilateral posterior cerebral vasogenic edema in one case	PRES
First author^*^	**Date of publication**	**Number of cases**	**Risk of Bias**	**Ocular manifestations or findings**	**Additional findings/Remarks**	**Diagnosis**
Agarwal${}^{†}$ ^[75]^	August 2020	115	High	30% of COVID-19 patients with brain MRI had leukoencephalopathy and/or cerebral microbleeds	
Cariddi^[76]^	June 2020	1	High	Blurred vision, altered mental status	Decreased nasolabial fold, tone and strength of the legs, and all deep tendon reflexes	PRES
Parauda${}^{†}$ ^[77]^	July 2020	4	High	Visually asymptomatic	Imaging showed cerebral vasogenic edema in four cases	PRES
Zhou^[78]^	June 2020	1	Medium	Bilateral subacute vision loss of both eyes. Pain with eye movements	Bilateral disc edema and venous congestion. Retinal perivenous hemorrhages in the right eye. Positive MOG-IgG. Both optic nerve enhancement	Optic neuritis associated with MOG-IgG
**Thromboembolic events**						
Dumitrascu^[79]^	May 2020	1	Medium	Sudden onset vision loss, no light perception	Retinal and optic disc edema, retinal exudates, attenuated retinal vessels, and absent macular cherry-red spot	Ophthalmic artery occlusion
Acharya^[80]^	June 2020	1	High	Sudden onset vision loss	Indistinct optic nerve margins, cherry red spot, significant retinal whitening	Central retinal artery occlusion
**Postmortem findings**						
Casagrande^[55]^	May 2020	14	Medium	Mentioned in the *Retinal findings* part	
Löffler^[86]^	June 2020	3	High	Case 1: optic atrophy Case 2: epiretinal gliosis, retinal paving-stone and spot bleeding, and drusen Case 3: AMD, macular atrophy, photoreceptor loss of outer retina, choroid thinning, and pinguecula	Case 1: MS Case 2: diabetic and hypertensive Case 3: Parkinsonism
Bayyoud^[87]^	June 2020	5	Medium	No viral RNA in samples of corneal stroma, endothelium and epithelium, conjunctival fluid swabs, bulbar-limbal conjunctiva, anterior chamber fluid	
Bayyoud^[88]^	June 2020	1	Low	No viral RNA in conjunctival fluid swabs, bulbar conjunctiva, corneal epithelium, stroma, and endothelium, anterior chamber fluid, lens, iris, vitreous, retina, uvea, sclera, and optic nerve	
Fuest^[89]^	July 2020	23	Medium	No viral RNA in conjunctival swabs	
*The studies are categorized based on the section of the eye involved in a study ${}^{†}$In these studies, the patients did not have any ophthalmologic findings or symptoms; however, due to the presence of ocular manifestations in some patients with PRES and leukoencephalopathy, the studies were included INO, internuclear ophthalmoplegia; GD1b-IgG antibody, antibody against ganglioside complex GD1b; MOG, Myelin Oligodendrocyte Glycoprotein; PRES, posterior reversible leukoencephalopathy; MFS, Miller Fisher syndrome; N/A, not available						

#### Episcleral involvement

A case of episcleritis was reported in Spain. A woman came with cough, myalgia, anosmia, and ageusia and had a positive PCR result for COVID-19. After these symptoms were resolved, she came to the ophthalmologic clinic with red eye, foreign-body sensation, epiphora, and photophobia, and nodular episcleritis was diagnosed. Besides, a study from Turkey revealed a 2.2% prevalence of episcleritis in COVID-19 patients. It was also shown that episcleritis was associated with a higher D-dimer level.^[25]^ The relationship between episcleritis and other viruses such as Ebola, HBV, HCV, and herpes zoster and immune-vascular factors and thrombotic complications of COVID-19 have risen the suspicion of the role of COVID-19 in developing episcleritis.^[39]^

#### Glaucoma and uveitis

A case of bilateral anterior uveitis following multisystem inflammatory syndrome in a COVID-19 patient has been reported so far.^[40]^ In addition, the involvement of uvea in other animals has been reported by other coronaviruses.^[41,42]^

To the best of our knowledge, no study has reported glaucoma as a presentation of COVID-19 yet. However, herpes virus detection in the trabecular meshwork and the following trabeculitis can result in intraocular pressure (IOP) elevation.^[43]^ Cytomegalovirus (CMV)-related anterior uveitis and the following rise of IOP in immunocompetent patients have been reported before, which is accompanied by the detection of CMV-DNA in aqueous humor.^[44,45,46,47]^ Hypertensive anterior uveitis can present with acute, recurrent, or chronic symptoms. By knowing the expression of ACE 2 receptor in aqueous humor^[16]^ and the role of CMV, herpes simplex virus, and Ebola virus in developing uveitic glaucoma, we can hypothesize the possible role of SARS-CoV-2 in developing, exacerbation, or recurrence of glaucoma.^[43,44,48]^ Therefore, it is not far from the mind that there will be more reports of uvea involvement or glaucoma progression in the future. If so, ophthalmologists should consider COVID-19 infection in patients with suspicious symptoms of SARS-CoV-2 and recurrent rise of IOP.

#### Ophthalmoparesis and nerve palsy

Dinkin et al reported two cases with ophthalmoparesis after their COVID-19 PCR became positive. In the first case, the patient developed areflexia, third nerve palsy of the left eye, and sixth nerve palsy of the right eye, resulting from immune response to the virus or direct invasion of the virus and the following demyelination process.^[49]^ The second case presented with diplopia, abduction deficit, and optic nerve sheath enhancement of the right eye, suggesting optic nerve involvement. However, whether optic nerve involvement resulted from CNS invasion of the virus or it was only a coincidence is not clear.^[50,51]^ In spite of the intact abducens nerve in MRI, sixth nerve palsy could not be ruled out in this case.

In Falcone et al's study, a patient developed diplopia and abduction deficit three days after the onset of the respiratory symptoms. MRI findings showed lateral rectus atrophy and denervation, all of which favored complete abducens nerve palsy.^[52]^ Another study revealed bilateral trochlear nerve palsy in a COVID-19 patient with evidence of cerebral vasculitis in brain MRI.^[53]^ Overall, all of the aforementioned cranial nerve involvements can be the spectrum of neurological manifestations of COVID-19, caused by either direct involvement of the CNS, excessive cytokine release, or endothelial dysfunction.^[54]^

#### Retinal findings

A study on retinal biopsies of 14 deceased patients with COVID-19 showed positivity of SARS-CoV-2 in three (21%) of them.^[55]^ It is in line with the expression of ACE 2 receptor in the human retina.^[15]^ Another study evaluated retinal findings in COVID-19 patients. It revealed hyperreflective lesions at the level of ganglion cell and inner plexiform layers. Besides, four patients had microhemorrhages and cotton wool spots. Other ophthalmologic investigations, including OCT angiography and ganglion cells complex analysis, visual acuity, and pupillary reflexes, were normal.^[56]^ Due to the previous reports of retinitis in animals and these two reports,^[57,58]^ the involvement of retina by SARS-CoV-2 is expectable. Interestingly, a patient was diagnosed with retinal vasculitis without any ophthalmologic symptoms.^[59]^ However, because of the absence of a control group and low sample size in this study, it is hard to differentiate whether SARS-CoV-2 or incidental findings cause these findings.

Another study compared OCT findings of optic nerve before and after COVID-19 infection in eight eyes of five patients. This study revealed an overall increase in RNFLT in seven of eight eyes, which can result from optic nerve inflammation.^[60]^ Aggravation of retinal lesions in diabetic retinopathy and papillophlebitis were also attributed to SARS-CoV-2-induced cytokine storm and inflammatory process in two studies.^[61,62]^

In addition to the direct involvement of the retina by SARS-CoV-2, COVID-19 can alter the pattern of some retinal diseases. Studies showed that COVID-19 lockdown was associated with less or delayed presentation and diagnosis of retinal detachment^[63]^ and a higher prevalence of proliferative vitreoretinopathy, accompanied by a lower response to surgery.^[64,65]^ Summaries of the retinal, neuro-ophthalmologic, thromboembolic, and postmortem findings are presented in Table 2.

#### Neuro-ophthalmologic manifestations

Like SARS-CoV, CNS involvement is seen in COVID-19 infection due to the expression of ACE2 receptor in nervous tissue and high affinity of SARS-CoV and SARS-CoV-2 with this receptor.^[66,67]^ Guillain-Barré syndrome in COVID-19 patients has been reported in several studies.^[51,68,69]^ Also, Miller Fisher syndrome, a variant of Guillain-Barré syndrome presenting with ophthalmoplegia has been observed in COVID-19 infection.^[70,71]^ In Gutiérrez-Ortiz et al's study, the first patient presented with fascicular oculomotor palsy and internuclear ophthalmoparesis of the right eye, and the second patient had bilateral abducens nerve palsy. Both of them had albuminocytologic dissociation, areflexia, and positive oropharyngeal PCR swab test, and COVID-19 was confirmed. While the first patient was a Miller Fisher syndrome case, the second was diagnosed as polyneuritis cranialis.^[70]^ In another study, a case with a previous history of strabismus of the left eye presented with left oculomotor nerve palsy, and after workup, Miller Fisher syndrome was diagnosed. The nasopharyngeal PCR test was positive for COVID-19. MRI showed significant enlargement and enhancement of the third cranial nerve. Later, the patient manifested with bilateral abducens nerve palsy.^[71]^

In another study conducted by Kaya et al, a 38-year-old man was admitted due to COVID-19 developing with sudden onset vision loss. Brain MRI findings showed vasogenic edema similar to what is observed in PRES. The exact association between SARS-CoV-2 and PRES and the etiology of PRES is not clear in this study. However, due to the neurotropism of SARS-CoV-2,^[72]^ it is thought that PRES can be the result of direct invasion of the virus and the inflammatory process in the setting of COVID-19 infection.^[73]^ It should be mentioned that several other studies reported PRES as a rare presentation of COVID-19, especially in severely ill patients.^[74,75,76,77]^ The severity of vision loss varies significantly in these studies, ranging from blurred vision to severe vision loss detecting only hand motions.

We also found a report of optic neuritis associated with the Myelin Oligodendrocyte Glycoprotein (MOG) antibody and COVID-19 infection, which can be another aspect of the neuro–ophthalmologic manifestations of COVID-19.^[78]^

#### Thromboembolic event

Dumitrascu et al presented the first ocular vascular complication of COVID-19. A 48-year-old obese man with severe COVID-19 infection and following upper and lower extremities DVTs was discharged from the hospital. Firstly, enoxaparin was administered for him, but later, it was changed to apixaban. One day after the apixaban start, he developed sudden onset vision loss of right eye without light perception and obvious relative afferent pupillary defect. Based on this history and a funduscopic examination, incomplete ophthalmic artery occlusion (OAO) was diagnosed.^[79]^ A case of central retinal artery occlusion was also reported in a patient with confirmed COVID-19 infection. Interestingly, contrary to the former case, no previous history of thrombotic events and anticoagulant consumption were reported for this patient.^[80]^

Due to the hypercoagulability state caused by COVID-19 infection,^[81,82,83]^ ophthalmologists should consider SARS-CoV-2 as a possible cause of OAO in patients with acute vision loss, especially in ones with underlying hypercoagulability state or thrombophilia. Also, more studies are needed to determine the optimal anticoagulant in COVID-19 patients to prevent such complications, especially in those with a hypercoagulable state.^[84,85]^

#### Post-mortem findings

In Löffler et al's study, three patients were evaluated for postmortem ocular findings. The findings were mainly due to their underlying diseases and conditions. For example, optic atrophy was found in a multiple sclerosis patient, and epiretinal gliosis, retinal paving-stone and spot bleeding, and drusen were observed in a diabetic hypertensive case. The third case, a 95-year-old patient with Parkinsonism, presented with age-related macular degeneration (AMD), macular atrophy, photoreceptor loss of the outer retina, choroid thinning, and pinguecula; in addition, autolytic changes were seen in microscopic evaluation of three cases. In another study, three of 14 retinal samples were positive for SARS-CoV-2 RdRp-gene, E-gene, and Orf nCoV-gene-specific sequences, which is consistent with the existence of ACE 2 receptors in human retinal cells.^[15,55,86]^ However, due to the absence of data on examining these patients before COVID-19 infection, we cannot ensure that coronavirus did not have any role in the occurrence or aggravation of these presentations. Additionally, three other studies evaluated the SARS-CoV-2 positivity in different ocular tissues of 29 deceased patients, all of which were negative.^[87,88,89]^

Overall, it seems that there is a low risk of ocular tissue involvement by SARS-CoV-2. However, studies with more sample sizes are needed to confirm this assumption. These findings should be considered when designing ophthalmology practice guidelines and criteria for corneal donors in the COVID-19 era.

Other coronaviruses have been reported to cause pyogranulomatous anterior uveitis, optic neuritis, choroiditis with retinal detachment, and retinal vasculitis in animals.^[41,42]^ Due to the presence of ACE 2 receptors in the ciliary body, vitreous body, and Muller cells,^[90,91]^ there may be further reports of the involvement of ocular structures by SARS-CoV-2 not reported yet. It also prompts ophthalmologists to remain vigilant about ocular manifestations of COVID-19. Furthermore, adhering to eye hygiene principles not only reduces the risk of SARS-CoV-2 transmission through the eye, but also reduces the risk of dry eye, bacterial infections, and the following ocular disorders.

##  Financial Support and Sponsorship

This project was not funded or sponsored by any agency or organization.

##  Conflicts of interest

The authors have no competing interests to declare.
